# Formation, Structure, and Thermal Annealing Effects of Ordered Self-Assembled Monolayers of 4-Fluorobenzeneselenol on Au(111)

**DOI:** 10.3390/molecules30092057

**Published:** 2025-05-05

**Authors:** Sicheon Seong, Jin Wook Han, Gayeong Joo, Hyun Sun Sung, Hong Kyu Park, Jaegeun Noh

**Affiliations:** 1 Department of Chemistry, Hanyang University, 222 Wangsimni-ro, Seongdong-gu, Seoul 04763, Republic of Korea; ssc09122@gmail.com (S.S.); jwhan@hanyang.ac.kr (J.W.H.); cocoa_0509@naver.com (G.J.); 9340ghdrb@naver.com (H.S.S.); gustjsdl1525@naver.com (H.K.P.); 2 Research Institute for Convergence of Basic Science, Hanyang University, 222 Wangsimni-ro, Seongdong-gu, Seoul 04763, Republic of Korea

**Keywords:** self-assembled monolayers, 4-fluorobenzeneselenol, adsorption, structure, ordered phase, thermal annealing effect, scanning tunneling microscopy

## Abstract

The formation, surface structure, and thermal annealing effects of self-assembled monolayers (SAMs) via vapor deposition of 4-fluorobenzeneselenol (4-FBSeH) on Au(111) at room temperature were investigated using scanning tunneling microscopy (STM). The most prominent structural feature is that 4-fluorobenzeneselenolate (4-FBSe) SAMs on Au(111) are composed of numerous SAM-covered Au adatom islands, regardless of the deposition time. High-resolution STM observations revealed that the ordered phase of 4-FBSe SAMs was formed after very short deposition times of 30 s and 3 min, whereas the disordered phase was formed after long deposition times of 1 h and 24 h. The ordered phase can be described as a (4 × 2√3) structure, and the average areal molecular density of the SAMs was calculated to be 29.0 Å^2^/molecule, suggesting the formation of densely packed monolayers with a standing-up adsorption structure. Interestingly, after thermal annealing at 373 K for 30 min, the (4 × 2√3) ordered phase of the SAMs was transformed to randomly distributed, short, single-molecular rows ranging from several nanometers to approximately ten nanometers in length, which has not been observed previously in organic thiolate SAMs. The high-resolution STM results of this study can provide very meaningful information for understanding the formation, surface structure, and thermal annealing effects of 4-FBSe SAMs on Au(111).

## 1. Introduction

Organic molecules containing sulfur and selenium as anchoring groups can readily form well-ordered self-assembled monolayers (SAMs) through chemical reactions between the anchoring groups and metal surfaces [1,2,3,4,5,6,7]. SAMs provide a very useful means for engineering the surface and interface properties of metal surfaces by modifying the chemical structures of molecular backbones and anchoring groups of the adsorbates [8,9,10,11,12,13,14,15]. Due to these technical advantages, SAMs are widely used for various technical applications, such as biosensors [16], bioelectronics [17,18], batteries [19], solar cells [20,21], thermoelectric devices [22], and molecular electronics [14,18,23,24,25,26]. In particular, halo-substituted aromatic thiolate/selenolate SAMs on metals have drawn much attention for the fabrication of SAM-based electronic devices because they can readily tune the charge injection barrier of semiconductor and metal surfaces, resulting in great improvements in the performance of devices [10,21,23,26,27,28,29,30,31]. It has been reported that the structural order, molecular orientation, and surface coverage of SAMs significantly affect the charge transfer properties of molecular electronic devices [32,33,34]. The molecular orientation of SAMs affects the hybridization between the molecular orbital and metal surface, which can alter charge transfer properties in molecular devices [32,33]. Well-ordered SAMs can significantly improve device performance by minimizing electrical shorts and by enhancing the reproducibility of device performance [34]. Therefore, optimizing SAM preparation conditions to produce well-ordered SAMs and understanding their surface structures are very important for developing SAM-modified electronic devices.

Fluorine-substituted aromatic thiolate SAMs are frequently used to control the work function of metal surfaces [23,26,27,28,29,30]. The direction of molecular dipole moments of SAMs derived from tetrafluorobenzenethiol (TFBSH) and pentafluorobenzenethiol (PFBSH) has a significant impact on the structural order and thermal stability of monolayers, as well as work function change in metal surfaces [26]. Scanning tunneling microscopy (STM) observations showed that the adsorption of TFBSH generated well-ordered SAMs with a (2√3 × 8√2)R30° structure at 323 K, while the adsorption of PFBSH molecules on Au(111) generated nicely ordered SAMs with a c(2 × √3) structure at 348 K [26]. It was also reported that Au adatom islands and molecular arrangements of PFBS SAMs on Au(111) at room temperature (RT) were significantly changed as a function of immersion time [35]. In particular, the SAMs of 4-fluorobenzenethiol (4-FBSH) have been frequently used to tune the work function of gold surfaces [29,30,36]. The work function of gold surfaces was precisely controlled using binary SAMs formed by changing the mole fraction of octadecanethiol and 4-FBSH [36]. STM measurements showed that the phase transitions of 4-FBS SAMs on Au(111) from a disordered phase to an ordered phase occurred as a result of long-term storage of the pre-covered SAMs [37] or prolonged immersion at RT for 24 h [38]. Additionally, ordered domains of 4-FBS SAMs could be formed via vapor deposition after a long deposition time of 24 h at 323 K [39].

On the other hand, organic selenolate SAMs on gold surfaces have drawn much attention due to their strong binding affinity compared to thiolate SAMs, resulting in the formation of highly ordered SAMs with high electrochemical and thermal stabilities [3,7,40,41,42,43,44,45,46,47,48,49,50]. The reductive desorption peaks of aliphatic and aromatic selenolate SAMs on Au(111) appeared at higher negative potentials than those of thiolate analogues, suggesting that the strength of the Se–Au bond is stronger than that of the S–Au bond [3,40]. This result can be strongly supported by the result that alkanethiolate SAMs are displaced by alkaneselenol molecules in solution, but the reverse displacement process does not occur [41]. Thermal desorption spectroscopy (TDS) measurements showed that the main desorption peak of methaneselenolate species from SAMs on Au(111) via the cleavage of Se–Au bonds was observed at around at 416 K, while that of methanethiolate species from SAMs via the cleavage of S–Au bonds was observed at around 366 K [42]. This suggests that the thermal stability of selenolate SAMs is much higher than that of thiolate SAMs. STM observations revealed that aliphatic and aromatic selenolate SAMs on Au(111) consist of larger ordered domains with different packing structures compared to their thiolate analogues [42,43,44,45]. It was also revealed that the work function of a gold surface can be easily controlled by using the Se anchoring group of the SAM instead of the S anchoring group [31,45]. Interestingly, alkyl selenolate SAM-based electronic devices were shown to have significantly longer lifetimes compared to thiolate SAM-based devices [50]. Meanwhile, despite numerous studies on fluorine-substituted aromatic thiolate SAMs on gold surfaces [23,26,27,28,29,30,35,36,37,38,39], there have been few reports describing the surface and interface properties of fluorine-substituted aromatic selenolate SAMs [31]. It has been shown that the formation, surface structure, and electronic properties of pentafulobenzeneselenolate (PFBSe) SAMs on Au(111) [31] are completely different from those of PFBS SAMs [26,35]. In contrast to many studies on 4-FBS SAMs, to date, no results have been reported describing the formation, structure, and thermal annealing effects of 4-fluorobenzeneselenolate (4-FBSe) SAMs on Au(111) from 4-fluorobenzeneselenol (4-FBSeH).

**Figure 1:**
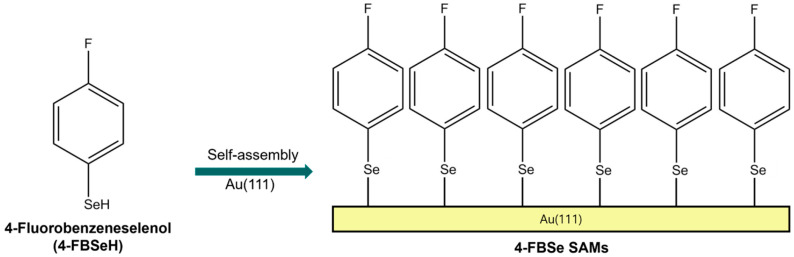
Chemical structure of 4-fluorobenzeneselenol (4-FBSeH) and the formation of 4-FBSe SAMs on Au(111) by the adsorption of 4-FBSeH molecules from vapor deposition.

In this study, to understand these issues of 4-FBSe SAMs, we investigated 4-FBSe SAM samples on Au(111) formed via ambient-pressure vapor-phase deposition at RT using STM. Figure 1 shows the molecular structure of 4-FBSeH and a schematic diagram showing the formation of SAMs on Au(111) by adsorption of 4-FBSeH molecules. Here, we report the first STM results showing a structural transition of 4-FBSe SAMs on Au(111) from a (4 × 2√3) ordered to a disordered phase with increasing deposition time. Furthermore, we also observed a very unique structural transition of the SAMs after thermal annealing at 373 K for 30 min, which has never been observed for thiolate SAMs.

## 2. Results and Discussion

### 2.1. The Formation and Surface Structure of 4-FBSe SAMs on Au(111) from Vapor Deposition

**Figure 2:**
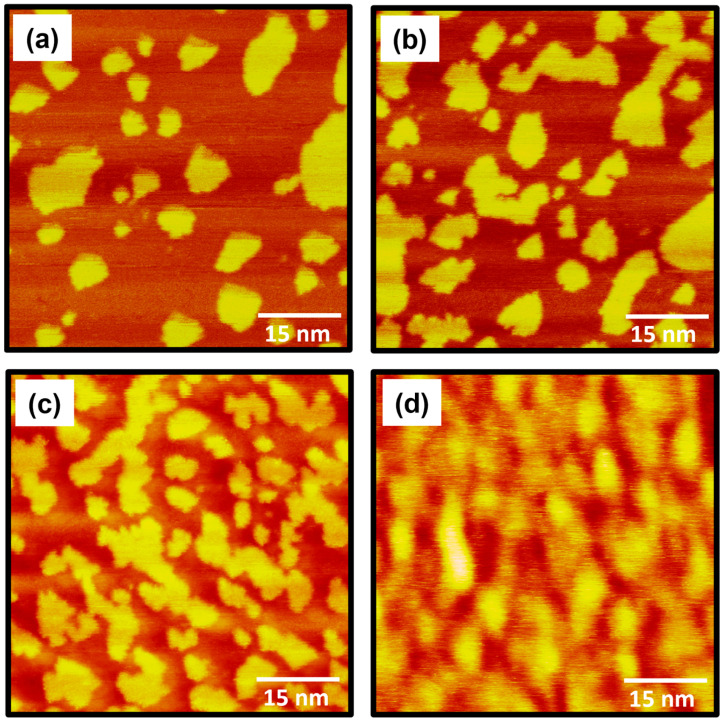
STM images showing the surface morphology of 4-FBSe SAMs on Au(111) formed via vapor deposition at RT as a function of deposition time: (**a**) 30 s, (**b**) 3 min, (**c**) 1 h, and (**d**) 24 h. The scan size and imaging parameters of all STM images are 60 nm × 60 nm, *I_t_* = 0.3 nA, and *V_b_* = 500 mV.

The STM images in Figure 2 show the very unique surface morphology of 4-FBSe SAMs on Au(111) formed after vapor deposition for (a) 30 s, (b) 3 min, (c) 1 h, and (d) 24 h at RT. The most striking structural features in the images are the existence of bright islands of various shapes protruding from the surface with a height of approximately 2.5 Å, corresponding to the monatomic step height of the Au(111) substrate. We would like to mention that the 2.5 Å height measured by STM corresponds to the apparent height, which depends on the applied bias voltage and does not directly reflect the actual topographic height. Therefore, these bright islands can be considered as SAM-covered Au adatom islands. Similar features have also been commonly observed for SAMs on Au(111) prepared from various aromatic thiols [26,34,35,37,38,51], benzenenselenol [52,53], bis(pentafluorophenyl)diselenide [31], and diphenyl diselenide [54]. These islands were usually observed for SAMs formed from aromatic molecules, in which anchoring groups containing sulfur and selenium were directly linked to the aromantic ring. For example, cyclohexanethiolate (or cyclohexaneselenolate) SAMs on Au(111) formed from cyclohexanethiol (or cyclohexaneselenol) having an aliphatic cyclohexyl ring do not contain such Au adatom islands [13,45], whereas benzenthiolate (or benzeneselenolate) SAMs having an aromatic benzene ring do contain such islands [34,52,53]. The formation of these islands is associated with the slow diffusion rate of thiolate– or selenolate–Au complexes generated during chemisorption of anchoring groups on gold surfaces, as discussed in the previous literature [51]. It is reasonable to assume that this slow diffusion rate during SAM formation is due to the interaction between the aromatic rings and the gold surfaces. Interestingly, despite the very short deposition time of 30 s, these islands were observed as shown in Figure 2a. The density of these islands appears to increase as the deposition time increases to 3 min and 1 h (Figure 2b,c). Finally, it was found that after a longer deposition of 24 h, these islands had a structure in which the islands merged with each other, and no clear boundaries were visible between the islands (Figure 2d). In contrast, it was found that many Au adatom islands that appeared in the as-prepared 4-fluorobenzenethiolate [37] or benzeneselenolate [54] SAMs could gradually disappear upon long-term storage for several days.

**Figure 3:**
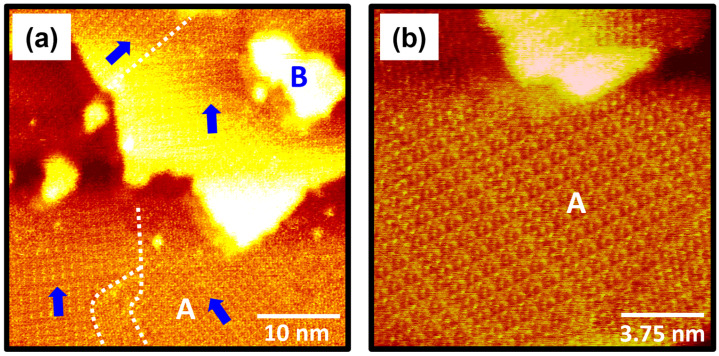
(**a**,**b**) STM images showing the large, ordered domains of 4-FBSe SAMs on Au(111) formed via vapor deposition at RT for 30 s. The ordered domains and Au adatom islands are labeled A and B, respectively. The directions of ordered domains are indicated by blue arrows and the dashed lines in the STM image correspond to domain boundaries. The scan sizes and imaging parameters of the STM images are (**a**) 40 nm × 40 nm, *I_t_* = 0.45 nA, and *V_b_* = 550 mV and (**b**) 15 nm × 15 nm, *I_t_* = 0.40 nA, and *V_b_* = 500 mV.

**Figure 4:**
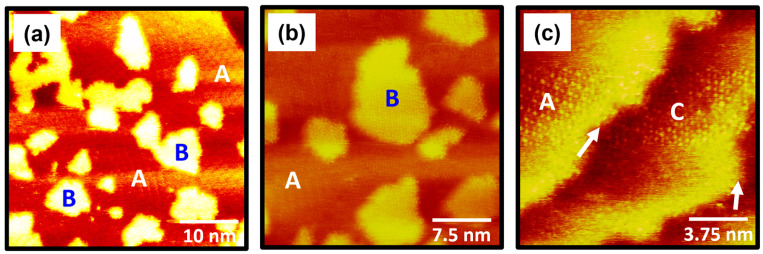
(**a**,**b**) STM images showing the ordered domains and many Au adatom islands of 4-FBSe SAMs on Au(111) formed via vapor deposition at RT for 3 min. The ordered domains and Au adatom islands are labeled A and B, respectively. (**c**) STM image showing ordered domains (labeled A) and randomly adsorbed domains (labeled C) of 4-FBSe SAMs around the step edges of Au(111). The step edges are indicated by white arrows in the STM image in (**c**). The scan sizes and imaging parameters of the STM images are (**a**) 40 nm × 40 nm, *I_t_* = 0.30 nA, and *V_b_* = 600 mV; (**b**) 30 nm × 30 nm, *I_t_* = 0.40 nA, and *V_b_* = 500 mV; and (**c**) 15 nm × 15 nm, *I_t_* = 0.45 nA, and *V_b_* = 500 mV.

The STM images in Figure 3 show the surface structure of 4-FBSe SAMs on Au(111) formed after vapor-phase deposition of 30 s at RT. STM observations clearly demonstrated that 4-FBSe SAMs consist of well-ordered domains (region A) and several bright Au adatom islands (region B), despite the very short deposition time of 30 s (Figure 4a). However, 4-FBSH molecules with sulfur anchoring groups form a disordered phase after a very short deposition time of 1 min at 323 K [39] and even after prolonged immersion in a 0.1 mM ethanol solution at RT for 24 h [37]. These results imply that 4-FBSeH molecules can form more easily ordered monolayers than thiol analogues. This is suggested to be because a stronger bond is formed between the Se anchoring group and the gold surface than between the S anchoring group and the gold surface, as demonstrated in previous studies [3,40]. The large-scale STM image (40 nm × 40 nm) in Figure 3a shows tri-directional ordered domains (indicated by blue arrows) with domain angles of 30°, 50°, and 80°. The dashed lines in the STM image correspond to domain boundaries. The observed domain orientations deviate from the three-fold symmetry of the Au(111) lattice, suggesting that there is no strong commensurable relationship between the SAM structure and the Au(111) lattice. Based on these results, it is suggested that the formation of ordered 4-FBSe SAMs is significantly influenced by van der Waals interactions between the 4-fluorobenzene backbones. Meanwhile, similar incommensurable domain structures were observed for arenethiolate SAMs with various domain angles of 10°, 50°, 60°, and 70° [55]. In contrast, commensurable SAM structures were frequently observed for alkanethiolate [51,56] and cyclohexylselenolate SAMs [45] with domain angles of 60° and 120°, suggesting that the SAM formation is mainly driven by the interaction between the anchoring group and the Au(111) surface. Previous studies have formalized that the formation of commensurate or incommensurate overlayers on solid substrates is mainly determined by the competition between molecule–molecule and molecule–substrate interactions [57]. The magnified STM image (15 nm × 15 nm) in Figure 3b clearly shows that well-ordered monolayers with a high periodicity are formed by the spontaneous adsorption of 4-FBSeH molecules from vapor deposition. The structural details of these SAMs will be discussed later.

After the deposition time increased from 30 s to 3 min, the surface features of 4-FBSe SAMs on Au(111) changed significantly, as shown in Figure 4. STM observations clearly revealed that the Au(111) surface was covered with the ordered SAMs (region A) and many bright Au adatom islands (region B) (Figure 4a). A striking structural feature is that the number of these adatom islands increases significantly with increasing deposition time. As shown in Figure 4a, the protruding adatom islands were observed to significantly interfere with STM imaging for obtaining molecular-scale information of the ordered monolayers (region A). Interestingly, the high-resolution STM image in Figure 4b clearly shows that the bright Au adatom islands are covered by the ordered SAMs rather than the bare Au(111) surface. This result may be supported by previous STM studies showing the presence of ordered domains on the surface of Au adatom islands [51]. The high-resolution STM image (15 nm × 15 nm) in Figure 4c shows that the Au(111) surface around the step edges (indicated by white arrows) is covered with ordered domains with high structural order (region A) and randomly adsorbed molecules (region C). This suggests that the step edges can significantly influence the formation and growth of ordered 4-FBSe SAMs on the Au(111) surface.

**Figure 5:**
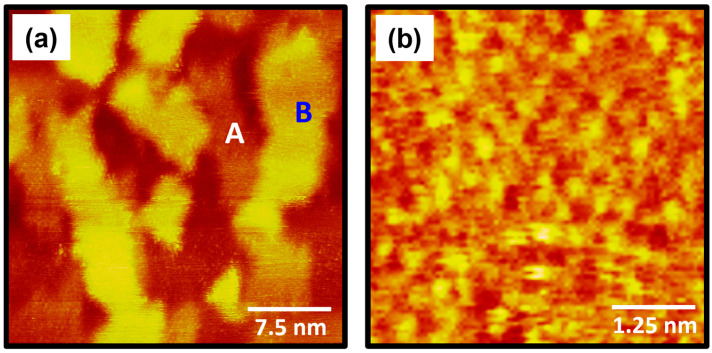
(**a**,**b**) STM images showing the randomly adsorbed structure of 4-FBSe SAMs on Au(111) formed via vapor deposition at RT for 1 h. The SAM-covered terraces and Au adatom islands are labeled A and B, respectively. The scan sizes and imaging parameters of the STM images are (**a**) 30 nm × 30 nm, *I_t_* = 0.40 nA, and *V_b_* = 500 mV and (**b**) 5 nm × 5 nm, *I_t_* = 0.40 nA, and *V_b_* = 500 mV.

After long-term deposition for 1 h, it was found that the surface morphology of 4-FBSe SAMs on Au(111) changed markedly. The STM image in Figure 5a showed that the large and uniform Au terraces covered with the ordered monolayers (region A, Figure 3 and Figure 4) became smaller, whereas the small-sized SAM-covered Au adatom islands (region B, Figure 4) grew larger with irregular shapes. The high-resolution STM image (5 nm × 5 nm) in Figure 5b showed the presence of randomly adsorbed molecules on the Au adatom islands. It is also worth noting that the terraces on the Au(111) surface almost disappeared after long-term deposition for 24 h, as shown in Figure 2d, resulting in the formation of a rough surface. Based on these STM studies, we clearly demonstrated that the surface morphology of the Au(111) surface was significantly changed by the adsorption of 4-FBSeH molecules on Au(111). The density of SAM-covered adatom islands increased with increasing deposition time, and these islands eventually coalesced to form a rough Au surface. We also found that very short deposition times are essential to fabricate 4-FBSe SAMs with uniform and highly ordered domains. These results are in conflict with those of 4-FBS SAMs, which showed that large, well-ordered 4-FBS SAMs without Au atomic islands were formed after long-term deposition on Au(111). It is strongly suggested that the Se anchoring group may be one of the important factors affecting the formation and growth of 4-FBSe SAMs and the surface morphology of the Au surface. In addition, the structural features of 4-FBSe SAMs are different from those of benzeneselenolate SAMs [54]. Therefore, we attribute this unusual structural behavior of 4-FBSe SAMs to the facile and rapid formation of 4-FBSe-Au complexes via chemisorption of 4-FBSeH molecules onto Au(111). The easy and fast formation of these complexes is attributed to the much weaker interactions between Au–Au bonds in the first Au layer, owing to the synergistic effect of the formation of strong Se–Au bonds and the strong electron-withdrawing effect of the fluorine substituents at the 4-position of the benzene ring. A similar scenario has been proposed in the previous literature to explain the significant differences in the formation and structure of thiolate and selenolate SAMs [45,58,59].

### 2.2. Molecular Packing Structure of 4-FBSe SAMs on Au(111)

**Figure 6:**
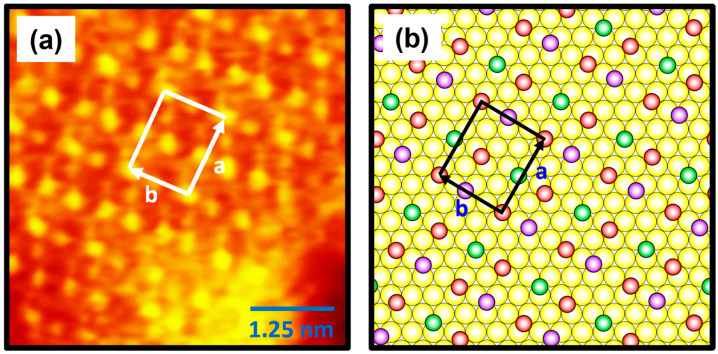
(**a**) High-resolution STM image of 4-FBSe SAMs on Au(111). (**b**) A proposed structural model of 4-FBSe SAMs on Au(111). The unit cell vectors (a and b) corresponding to the ordered phase are shown in (**a**,**b**). In the structural model, the red circles correspond to the bridge sites, the purple circles correspond to the three-fold hollow sites, and the green circles correspond to the sites between the atop and the three-fold hollow sites. Additionally, the yellow circles correspond to the Au atoms. The height of the molecules represented by the green circles was slightly (~0.1 Å) lower than that of the brightest molecules. The scan size and imaging parameters of the STM image are 5 nm × 5 nm, *I_t_* = 0.50 nA, and *V_b_* = 550 mV.

The molecularly resolved STM image (5 nm × 5 nm) in Figure 6a clearly shows the molecular arrangements of 4-FBSe SAMs on Au(111) with a high degree of structural order after vapor deposition for 30 s. Based on the high-resolution STM images in Figure 6a, the lattice parameters of rectangular unit cells containing four 4-FBSe molecules adsorbed on Au(111) were extracted: a = 11.6 ± 0.2 Å = 4a_h_ and b = 10.0 ± 0.2 Å = 2√3a_h_, where a_h_ represents the interatomic distance between Au atoms. Figure 6b shows the proposed structural model of 4-FBSe SAMs on Au(111). The ordered phase of 4-FBSe SAMs can be described as a (4 × 2√3) structure, which is completely different from the structure of 4-FBS SAMs with a (4 × √3)R30° phase formed from vapor deposition at 323 K for 24 h [39] or a (16 × √3) phase formed after long-term storage of the as-prepared SAMs for several days [37]. These results imply that the interaction between the selenium anchoring group and the Au(111) surface is one of the important parameters determining the packing structure of 4-FBSe SAMs. In the structural model, the brightest molecule was assumed to adsorb on the bridge sites (red circles) of the Au(111) lattice, and the darker molecule was assumed to adsorb on the three-fold hollow sites (purple circles). The height of the molecule represented by the green circle was slightly (~0.1 Å) lower than that of the brightest molecule. Thus, they were placed midway between the atop and the three-fold hollow sites. The average areal molecular density of 4-FBSe SAMs was calculated to be 29.0 Å^2^/molecule, which is very similar to that of 4-FBS SAMs with 28.9 Å^2^/molecule [39]. In addition, the closely packed benzeneselenolate SAMs with a standing-up adsorption structure also showed a very similar areal density of 28.5 Å^2^/molecule [53]. Compared with previous results [39,53], it is reasonable to assume that the ordered phase of 4-FBSe SAMs formed after a very short deposition of 30 s has densely packed monolayers with a standing-up adsorption structure.

### 2.3. Structural Changes in 4-FBSe SAMs on Au(111) After Thermal Annealing

**Figure 7:**
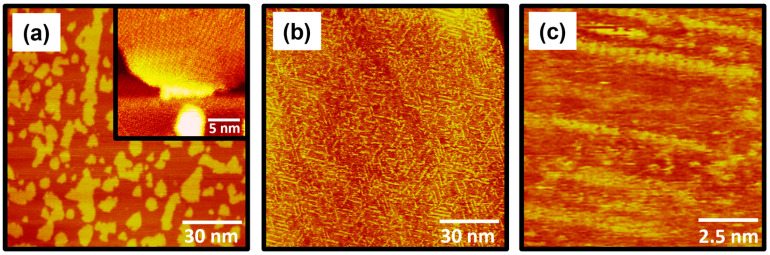
(**a**) STM image of pre-covered 4-FBSe SAMs on Au(111) formed via vapor deposition at RT for 30 s. The inset STM image (20 nm × 20 nm) shows that 4-FBSe SAMs consist of ordered phases and Au adatom islands. (**b**,**c**) STM images showing significant structural transitions of pre-covered 4-FBSe SAMs on Au(111) after thermal annealing at 373 K for 30 min. The scan sizes and imaging parameters of the STM images were (**a**) 120 nm × 120 nm, *I_t_* = 0.30 nA, and *V_b_* = 500 mV; (**b**) 120 nm × 120 nm, *I_t_* = 0.35 nA, and *V_b_* = 620 mV; and (**c**) 10 nm × 10 nm, *I_t_* = 0.52 nA, and *V_b_* = 550 mV.

Understanding the surface and interfacial properties of organic SAMs on metal surfaces depending on thermal treatment is a very important issue from both fundamental and applied perspectives [7,26,42,53,56,60,61,62,63,64,65,66]. In general, many STM studies have focused on investigating the molecular-scale structural changes in alkanethiolate SAMs as a function of thermal annealing time at high temperatures [60,61,62,63,64]. However, to date, no STM studies have been reported to elucidate the molecular-scale surface features of selenolate SAMs on Au(111) after thermal annealing. In this study, we investigated the surface morphology changes in 4-FBSe SAMs on Au(111) after thermal annealing at 373 K and for 30 min (Figure 7). Since it has been reported that long-range and well-ordered octanethiolate SAMs with few structural defects are formed after thermal annealing at 373 K and for 30 min [64], the same annealing conditions were used. It is well known that thermal treatment utilizing optimized conditions, such as annealing temperature and time, can significantly improve the quality of the structural order of alkanethiolate SAMs by reducing structural defects such as vacancy islands and domain boundaries and increasing the size of ordered domains resulting from the Ostwald ripening process [60,61,62,63,64]. However, we found that the structural changes in 4-FBSe SAMs due to thermal annealing were completely different from those in alkanethiolate SAMs. What is interesting is that a very unique structural change in the 4-FBSe SAMs was observed upon thermal annealing (Figure 7), which has not been previously observed in thiolate SAMs. After thermal annealing, the (4 × 2√3) ordered phase of the SAMs containing many Au adatom islands (Figure 7a) was changed into a mixed phase consisting of the disordered phase and randomly distributed, short, single-molecular rows with lengths ranging from several nanometers to approximately ten nanometers (Figure 7b). In addition, many Au adatom islands completely disappeared. The magnified STM image (10 nm × 10 nm) in Figure 7c shows individual molecules in a molecular row. The distance between molecules in the rows was measured to be 5 Å, which corresponds to √3 times the diameter of a gold atom. The reason why the very unique structural features of the selenolate SAMs are formed by thermal annealing is not yet clearly understand, but it is assumed that the rearrangement of the adsorbed molecules occurs in the process of many Au atom islands disappearing on the gold surface, and, as a result, the packing structure of the SAMs is completely changed. Based on these STM observations, we clearly demonstrated that the structural changes in selenolate SAMs due to thermal annealing are significantly different from those of thiolate SAMs.

## 3. Experimental Section

### 3.1. Chemicals and Preparation of Au(111) Substrates

4-FBSeH was synthesized from pure selenium and 1-bromo-4-fluorobenzene according to a previously reported method [67] and was confirmed by ^1^H-NMR. Au(111) substrates were prepared by thermal evaporation of gold onto freshly cleaved mica surfaces preheated to 570 K under ultra-high-vacuum conditions of about 10^−5^ Pa. STM observations revealed that the Au(111) substrates contained large, atomically flat terraces with sizes ranging from 100 to 400 nm.

### 3.2. Preparation of 4-FBSe SAMs

To understand the formation and growth processes of 4-FBSe SAMs on Au(111), SAMs were prepared in the vapor phase by placing Au(111) substrates in 3 mL V-vials containing 1 μL of pure 4-FBSeH neat liquid at RT as a function of deposition time. In this case, there should be no direct contact between the Au(111) substrate and the pure neat liquid in the vial during SAM formation. The vials were then tightly sealed with Teflon tape and kept at RT for the desired deposition times (30 s, 3 min, 1 h, and 24 h). The prepared SAM samples were carefully washed with pure ethanol to remove the physisorbed 4-FBSeH molecules on the SAM surfaces and were then dried under high-purity N_2_ gas flow before surface characterization.

### 3.3. STM Measurements

STM measurements were performed with a NanoScope E (Veeco, Santa Barbara, CA, USA) using commercially available Pt/Ir (80:20) tips. All STM images were obtained using the constant current mode in air at RT. For STM imaging, a bias voltage (*V_b_*) of 300 to 700 mV and tunneling currents (*I_t_*) of 0.30 and 0.60 nA were applied between the tip and the sample.

## 4. Conclusions

The formation, structure, and thermal annealing effects of 4-FBSe SAMs on Au(111) were investigated by STM. The most prominent structural feature is that 4-FBSe SAMs on Au(111) are composed of many SAM-covered Au adatom islands, regardless of vapor deposition time. The density of these islands was found to increase as the deposition time increased to 30 s, 3 min, and 1 h. Finally, after deposition for 24 h, the islands merged with each other and showed a rough structure without clear boundaries. STM observations showed that the ordered phase of 4-FBSe SAMs was formed via very short deposition times for 30 s and 3 min, whereas the disordered phase was formed after long deposition times of 1 h and 24 h. The ordered phase is assigned to a (4 × 2√3) structure, which is completely different from that of 4-FBS SAMs with a (4 × √3)R30° phase without Au adatom islands. The average areal molecular density of 4-FBSe SAMs was calculated to be 29.0 Å^2^/molecule, suggesting the formation of densely packed monolayers with a standing-up adsorption structure. Interestingly, after thermal annealing at 373 K for 30 min, the (4 × 2√3) ordered phase of the SAMs was changed into a mixed phase consisting of the disordered phase and randomly distributed, short, single-molecular rows with lengths ranging from several nanometers to approximately ten nanometers. The Se anchoring group and the fluorine substituent on the benzene ring were found to play an important role in the formation and growth of 4-FBSe SAMs. The molecular-scale STM results in this study provide new insights into the formation, surface structure, and thermal annealing effects of 4-FBSe SAMs on Au(111).

## Data Availability

The data presented in this study are available in the article.
